# “Warranted” Dental Treatment

**Published:** 1914-10-15

**Authors:** Henry Schwamm

**Affiliations:** The N. Y. Bar


### "WARRANTED" DENTAL TREATMENT.

BY HENRY SCHWAMM, D.D.S., LL. B., OF THE N. Y. BAR

Inexperienced dentists, and those who are forced by economic conditions, to build up a practice along lines of least resistance, among the poor and uneducated, sometimes insert in their stationery, or on their signs, the words, "All work guaranteed" or "All work guaranteed ten years."

They little realize the reponsibility they assume thereby, as is shown by the use of the word "guaranty," which is popularly understood to mean warranty. Whenever this question arises, as it does quite frequently, although mostly in inferior courts, the courts has no difficulty in finding that the word "guaranty" was intended to mean "warranty." Guaranty is "a contract by which one person is bound to another for the fulfillment of the promise or engagement of a third party," while warranty is an undertaking that a certain fact regarding the subject of a contract is what it has been represented to be, and relates to some agreement made ordinarily by the party who makes the warranty. But it is settled that when a dentist makes positive representations as to character, quality or durability of certain work, not mere matter of opinion or judgment, and the patient understands it as a warranty, and he relies upon it and is induced by it, the dentist is bound by his representations, no matter whether he intended it to be a warranty or not. The dentist is responsible for the language he uses, and he can not escape liability by claiming that he did not intend to convey the impression which his language was calculated to produce upon the mind of the patient. The principles governing contractual relations in the general business world will be enforced by the court in cases where dentists enter into contracts with patients, expressed or implied. When a buyer purchases an article whose true character he can not discover by any examination which it is practicable for him to make at the time, why may he not rely upon the positive representation of the seller as to its character, as well as to its quality and conditions? No acceptable distinction in principle can be offered in the representations made by a dentist and the ones made by another person, when those representations are understood by the patient, or the vendee, as the case may be, to constitute a warranty, and the latter rely on them. The giving of warranties is condemnable from every aspect. It is not merely unethical, but, considering the uncertain durability of dental work, it implies fraud in most cases. Relying on the promise, the patients acquire a false sense of security against dental trouble, become neglectful, instead of periodically visiting the dentist, and when the dental defects become apparent, seek redress in court. It is not uncommon to find persons holding dental warranties, using the same for fraudulent purposes against dentists, and, owing to the nature of the transaction, are mostly successful. We have now, in our office, three cases pending, in which dentists are the defendants, for giving warranties—one is little less than blackmail against the dentist, considering the negligence and lack of notice by the patient to the dentist; in the other two cases we defend the dentists more as a matter of duty than pleasure.—*Dental Outlook.*

